# Ruptured Renal Angiomyolipoma

**DOI:** 10.5334/jbsr.2932

**Published:** 2022-11-04

**Authors:** Sébastien Azzi, Patrice Jissendi, Fadi Tannouri

**Affiliations:** 1 CHU Saint Pierre de Bruxelles, BE; 2 CUB Hôpital Erasme, Brussels, BE

**Keywords:** angiomyolipoma, retroperitoneal haemorrhage, acute abdomen, renal tumour

## Abstract

**Teaching Point:** This case highlights the importance of extending the medical analysis to other areas with lumbar computed tomography, especially to the kidneys and the retroperitoneum.

## Case History

A 47-year-old female was admitted to the emergency with exacerbating left lumbar pain, irradiating to the inguinal region. The patient had no known medical or surgical history, other than chronical lower back pain. Laboratory workup showed no sign of inflammation or urine abnormalities, except for a drop of haemoglobin.

**Figure 1:**
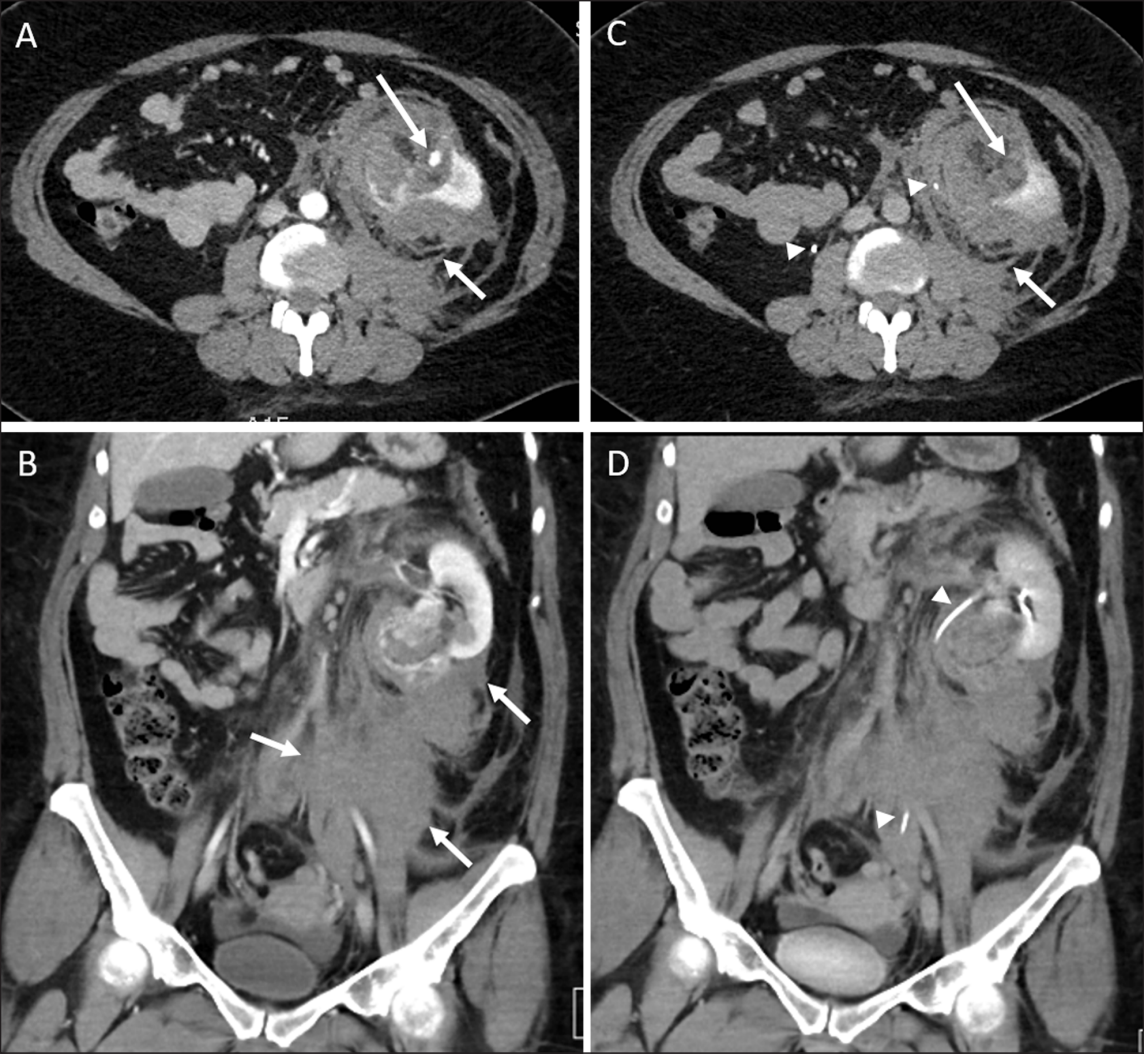


**Figure 2:**
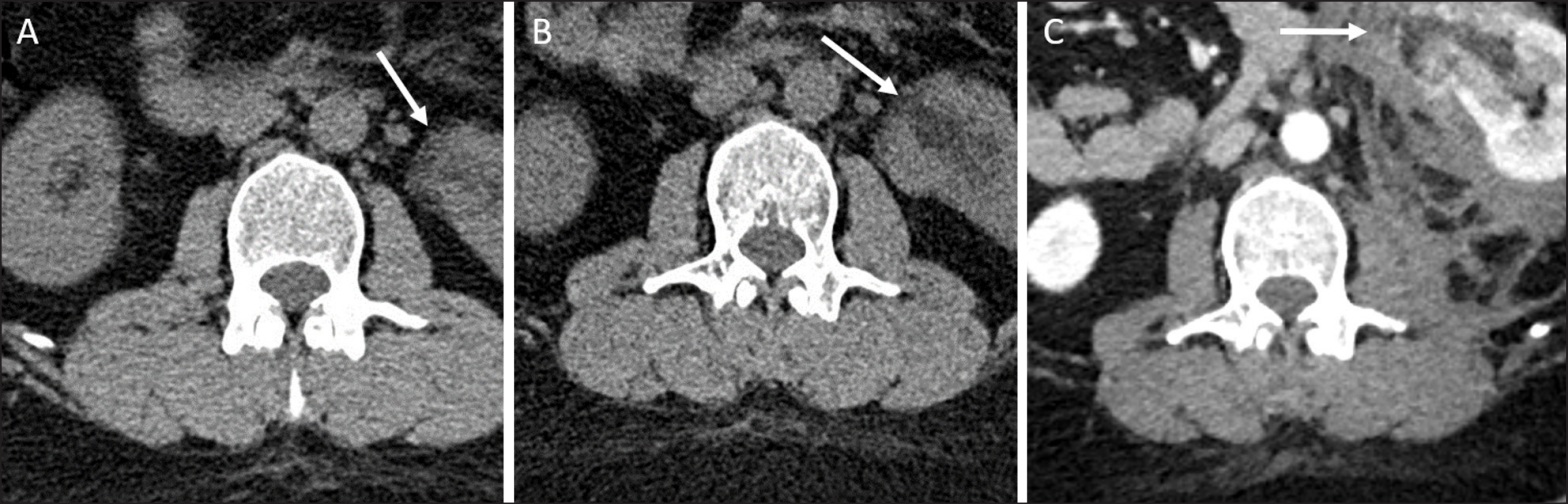


A double-phase (late arterial and excretory phase) contrast-enhanced computed tomography (CT) was performed. The late arterial phase showed a 54 mm heterogeneous fat containing mass involving the lower pole of the left kidney and most likely the cause for intra- and retroperitoneal fluid collections (Figure 1). Leakage of contrast within the lesion was observed, suggesting active bleeding (Figure 1A, B). At the delayed phase, the collecting system was compressed by the hematoma (Figure 1C, D). The location of the lesion, the imaging features, including non-enhancing (fat) and enhancing components (solid tissue and vessels), and the fluid collections infiltrating the retroperitoneal space, suggested the diagnosis of ruptured bleeding renal angiomyolipoma. A retrospective review of the lumbar spinal CT study, prior to the current exam (2014 and 2018), showed that the renal lesion was present 8 years prior, but only partly visible because the retroperioneal space was largely out of the field of view (Figure 2).

**Figure 3:**
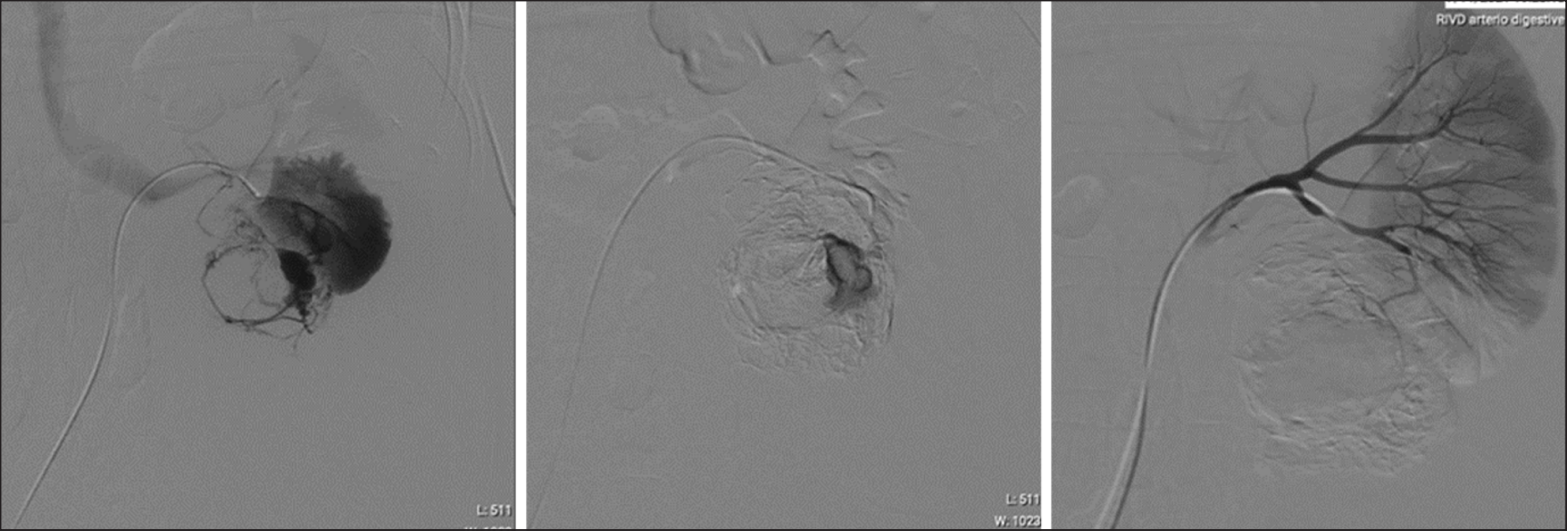


Given the location and the presence of active bleeding, it was decided to treat the patient by selective renal artery embolization rather than nephrectomy (Figure 3).

## Discussion

Renal angiomyolipoma (AML) is a rare benign tumour, composed of thick-walled vessels, smooth muscle elements and fatty tissue. The diagnosis of sporadic AML is incidental during cross-sectional imaging in the majority of cases because the lesion is often asymptomatic and small in size. Rarely, a bulky AML can present as a palpable mass. A tumour with a diameter larger than 4 cm is more likely to develop aneurysms more susceptible to bleeding [1]. Acute AML bleeding manifests as the Lenk triad, which includes acute flank pain, abdominal tenderness, and signs of internal bleeding such as haematuria. The acute haemorrhage can be life threatening and then require urgent surgical treatment.

Treatment options have to be discussed on an individual base. Selective embolization of AML is a safe and minimally invasive procedure with very few complications. It allows to achieve fast curation of the bleeding in the majority of patients. In addition, the acquired reduction in tumour size is long-lasting and significant. Partial nephrectomy, or more rarely, nephrectomy has a lower incidence of recurrence but a higher complication rate as compared with embolization and should be performed in selected patients only.
